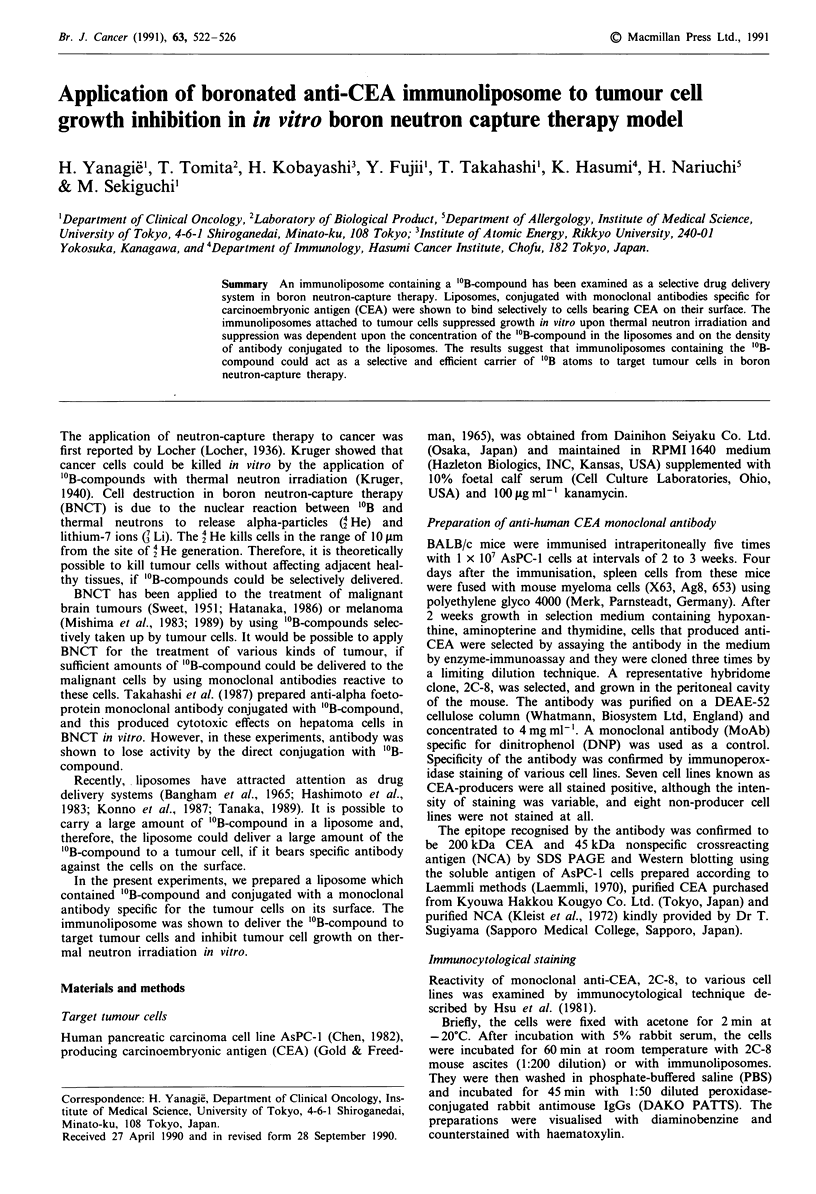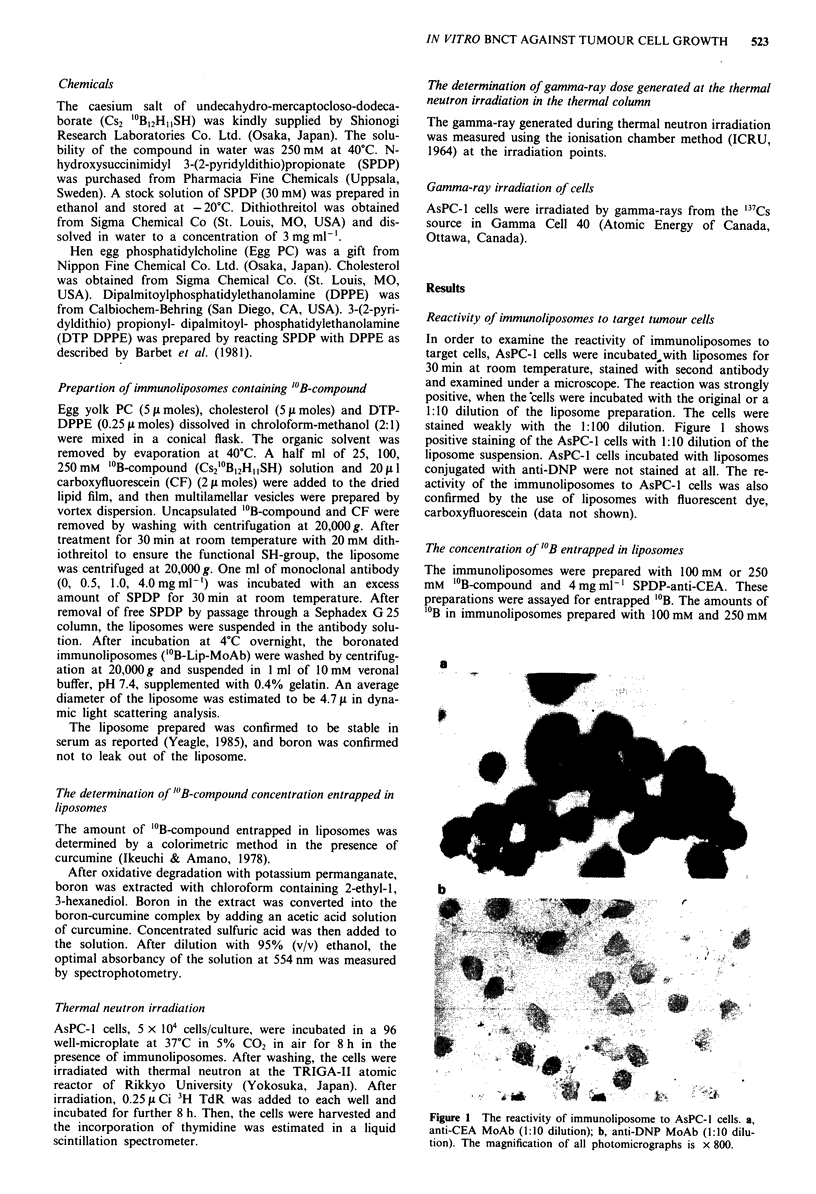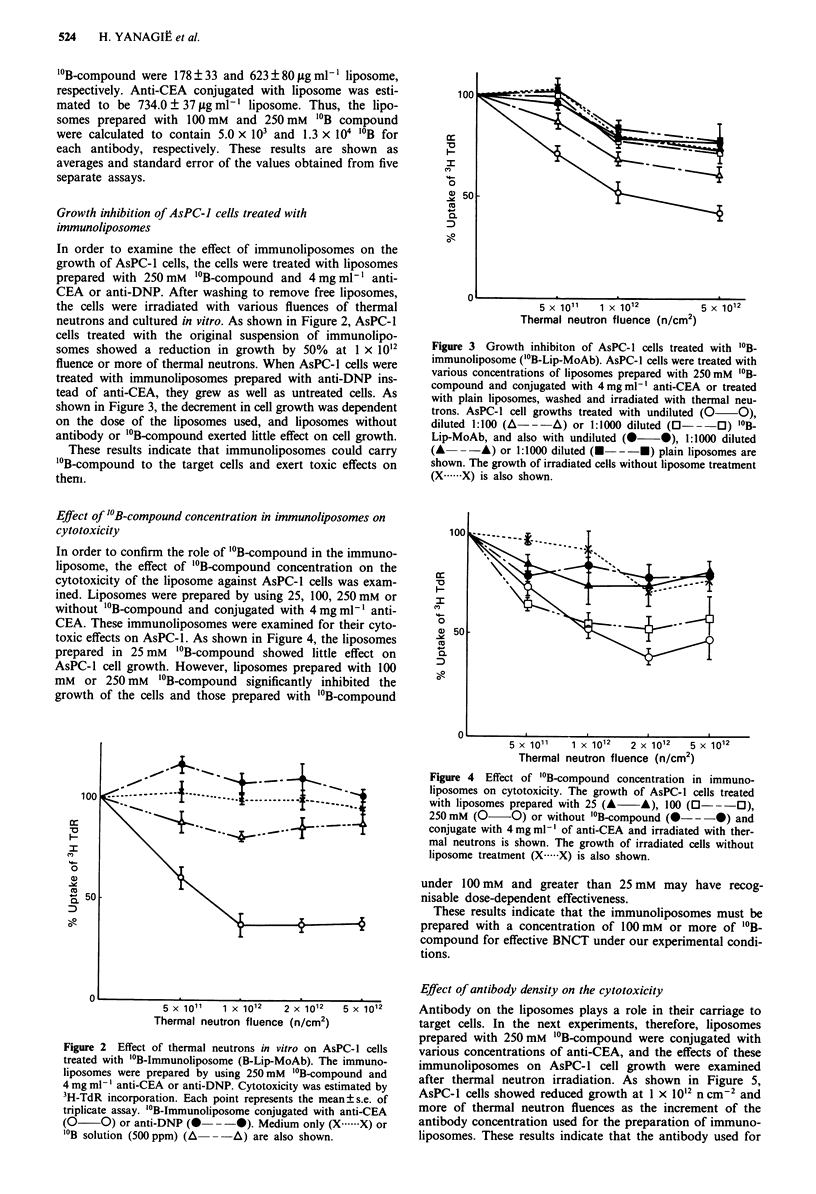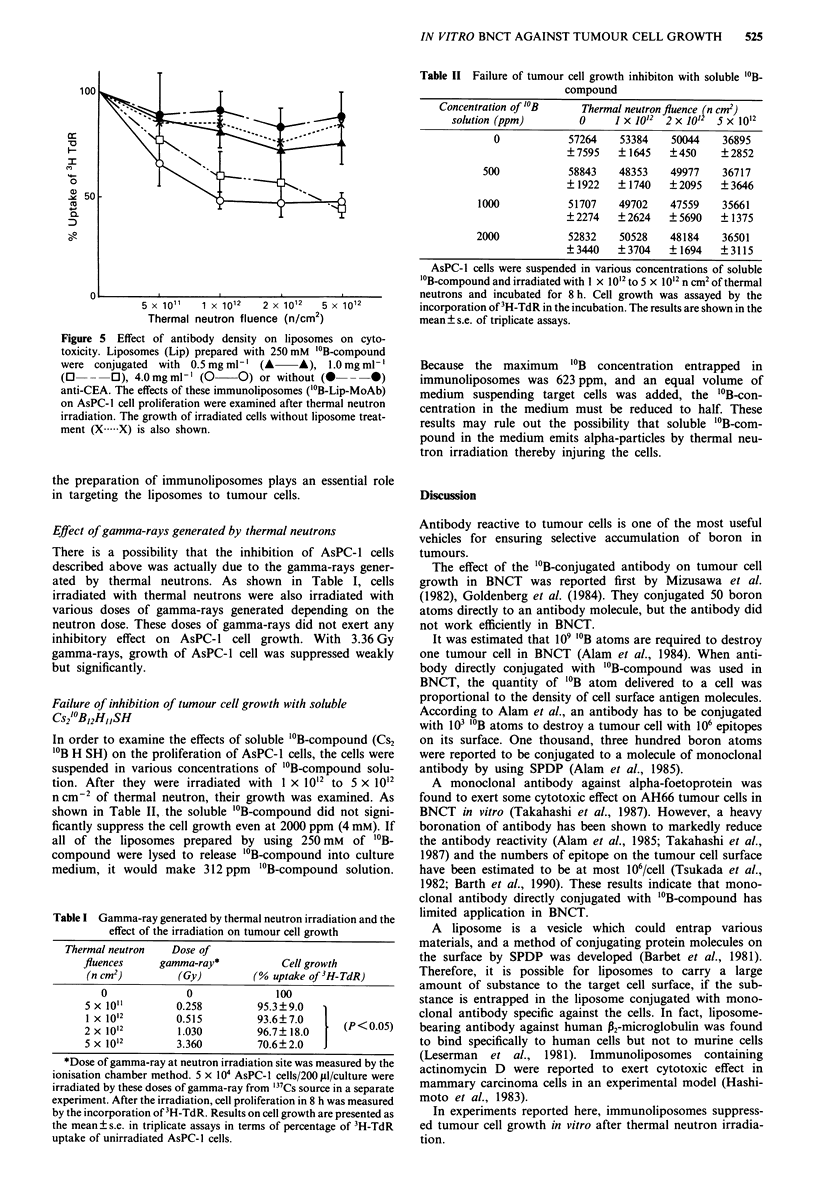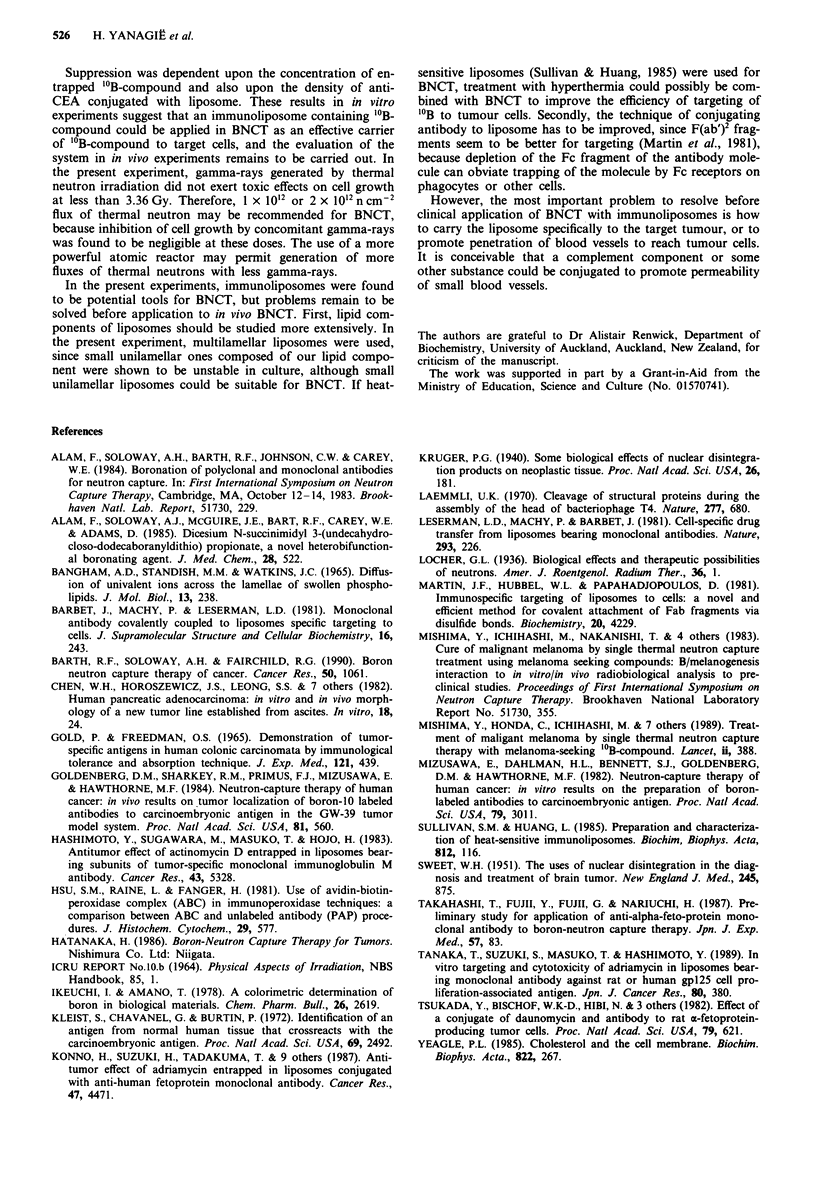# Application of boronated anti-CEA immunoliposome to tumour cell growth inhibition in in vitro boron neutron capture therapy model.

**DOI:** 10.1038/bjc.1991.124

**Published:** 1991-04

**Authors:** H. Yanagië, T. Tomita, H. Kobayashi, Y. Fujii, T. Takahashi, K. Hasumi, H. Nariuchi, M. Sekiguchi

**Affiliations:** Department of Clinical Oncology, University of Tokyo, Japan.

## Abstract

**Images:**


*Br. J. Cancer* (1991), **63**, 522–526 © Macmillan Press Ltd., 1991

### Application of boronated anti-CEA immunoliposome to tumour cell growth inhibition in *in vitro* boron neutron capture therapy model

H. Yanagië[1], T. Tomita[2], H. Kobayashi[3], Y. Fujii[1], T. Takahashi[1], K. Hasumi[4], H. Nariuchi[5] & M. Sekiguchi[1]

[1]*Department of Clinical Oncology,* [2]*Laboratory of Biological Product,* [5]*Department of Allergology, Institute of Medical Science, University of Tokyo, 4-6-1 Shiroganedai, Minato-ku, 108 Tokyo;* [3]*Institute of Atomic Energy, Rikkyo University, 240-01 Yokosuka, Kanagawa, and* [4]*Department of Immunology, Hasumi Cancer Institute, Chofu, 182 Tokyo, Japan.*

**Summary** An immunoliposome containing a $^{10}$B-compound has been examined as a selective drug delivery system in boron neutron-capture therapy. Liposomes, conjugated with monoclonal antibodies specific for carcinoembryonic antigen (CEA) were shown to bind selectively to cells bearing CEA on their surface. The immunoliposomes attached to tumour cells suppressed growth *in vitro* upon thermal neutron irradiation and suppression was dependent upon the concentration of the $^{10}$B-compound in the liposomes and on the density of antibody conjugated to the liposomes. The results suggest that immunoliposomes containing the $^{10}$B-compound could act as a selective and efficient carrier of $^{10}$B atoms to target tumour cells in boron neutron-capture therapy.

The application of neutron-capture therapy to cancer was first reported by Locher (Locher, 1936). Kruger showed that cancer cells could be killed *in vitro* by the application of $^{10}$B-compounds with thermal neutron irradiation (Kruger, 1940). Cell destruction in boron neutron-capture therapy (BNCT) is due to the nuclear reaction between $^{10}$B and thermal neutrons to release alpha-particles ($^4_2$He) and lithium-7 ions ($^7_3$Li). The $^4_2$He kills cells in the range of 10 μm from the site of $^4_2$He generation. Therefore, it is theoretically possible to kill tumour cells without affecting adjacent healthy tissues, if $^{10}$B-compounds could be selectively delivered.

BNCT has been applied to the treatment of malignant brain tumours (Sweet, 1951; Hatanaka, 1986) or melanoma (Mishima *et al.*, 1983; 1989) by using $^{10}$B-compounds selectively taken up by tumour cells. It would be possible to apply BNCT for the treatment of various kinds of tumour, if sufficient amounts of $^{10}$B-compound could be delivered to the malignant cells by using monoclonal antibodies reactive to these cells. Takahashi *et al.* (1987) prepared anti-alpha foetoprotein monoclonal antibody conjugated with $^{10}$B-compound, and this produced cytotoxic effects on hepatoma cells in BNCT *in vitro*. However, in these experiments, antibody was shown to lose activity by the direct conjugation with $^{10}$B-compound.

Recently, liposomes have attracted attention as drug delivery systems (Bangham *et al.*, 1965; Hashimoto *et al.*, 1983; Konno *et al.*, 1987; Tanaka, 1989). It is possible to carry a large amount of $^{10}$B-compound in a liposome and, therefore, the liposome could deliver a large amount of the $^{10}$B-compound to a tumour cell, if it bears specific antibody against the cells on the surface.

In the present experiments, we prepared a liposome which contained $^{10}$B-compound and conjugated with a monoclonal antibody specific for the tumour cells on its surface. The immunoliposome was shown to deliver the $^{10}$B-compound to target tumour cells and inhibit tumour cell growth on thermal neutron irradiation *in vitro*.

### Materials and methods

### *Target tumour cells*

Human pancreatic carcinoma cell line AsPC-1 (Chen, 1982), producing carcinoembryonic antigen (CEA) (Gold & Freedman, 1965), was obtained from Dainihon Seiyaku Co. Ltd. (Osaka, Japan) and maintained in RPMI 1640 medium (Hazleton Biologics, INC, Kansas, USA) supplemented with 10% foetal calf serum (Cell Culture Laboratories, Ohio, USA) and 100 μg ml$^{-1}$ kanamycin.

### *Preparation of anti-human CEA monoclonal antibody*

BALB/c mice were immunised intraperitoneally five times with 1 × 10$^7$ AsPC-1 cells at intervals of 2 to 3 weeks. Four days after the immunisation, spleen cells from these mice were fused with mouse myeloma cells (X63, Ag8, 653) using polyethylene glyco 4000 (Merk, Parnsteadt, Germany). After 2 weeks growth in selection medium containing hypoxanthine, aminopterine and thymidine, cells that produced anti-CEA were selected by assaying the antibody in the medium by enzyme-immunoassay and they were cloned three times by a limiting dilution technique. A representative hybridome clone, 2C-8, was selected, and grown in the peritoneal cavity of the mouse. The antibody was purified on a DEAE-52 cellulose column (Whatmann, Biosystem Ltd, England) and concentrated to 4 mg ml$^{-1}$. A monoclonal antibody (MoAb) specific for dinitrophenol (DNP) was used as a control. Specificity of the antibody was confirmed by immunoperoxidase staining of various cell lines. Seven cell lines known as CEA-producers were all stained positive, although the intensity of staining was variable, and eight non-producer cell lines were not stained at all.

The epitope recognised by the antibody was confirmed to be 200 kDa CEA and 45 kDa nonspecific crossreacting antigen (NCA) by SDS PAGE and Western blotting using the soluble antigen of AsPC-1 cells prepared according to Laemmli methods (Laemmli, 1970), purified CEA purchased from Kyouwa Hakkou Kougyo Co. Ltd. (Tokyo, Japan) and purified NCA (Kleist *et al.*, 1972) kindly provided by Dr T. Sugiyama (Sapporo Medical College, Sapporo, Japan).

### *Immunocytological staining*

Reactivity of monoclonal anti-CEA, 2C-8, to various cell lines was examined by immunocytological technique described by Hsu *et al.* (1981).

Briefly, the cells were fixed with acetone for 2 min at −20°C. After incubation with 5% rabbit serum, the cells were incubated for 60 min at room temperature with 2C-8 mouse ascites (1:200 dilution) or with immunoliposomes. They were then washed in phosphate-buffered saline (PBS) and incubated for 45 min with 1:50 diluted peroxidase-conjugated rabbit antimouse IgGs (DAKO PATTS). The preparations were visualised with diaminobenzine and counterstained with haematoxylin.

Correspondence: H. Yanagië, Department of Clinical Oncology, Institute of Medical Science, University of Tokyo, 4-6-1 Shiroganedai, Minato-ku, 108 Tokyo, Japan.
Received 27 April 1990 and in revised form 28 September 1990.

*Chemicals*

The caesium salt of undecahydro-mercaptocloso-dodecaborate ($Cs_2$ $^{10}B_{12}H_{11}SH$) was kindly supplied by Shionogi Research Laboratories Co. Ltd. (Osaka, Japan). The solubility of the compound in water was 250 mM at 40°C. N-hydroxysuccinimidyl 3-(2-pyridyldithio)propionate (SPDP) was purchased from Pharmacia Fine Chemicals (Uppsala, Sweden). A stock solution of SPDP (30 mM) was prepared in ethanol and stored at −20°C. Dithiothreitol was obtained from Sigma Chemical Co (St. Louis, MO, USA) and dissolved in water to a concentration of 3 mg $ml^{-1}$.

Hen egg phosphatidylcholine (Egg PC) was a gift from Nippon Fine Chemical Co. Ltd. (Osaka, Japan). Cholesterol was obtained from Sigma Chemical Co. (St. Louis, MO, USA). Dipalmitoylphosphatidylethanolamine (DPPE) was from Calbiochem-Behring (San Diego, CA, USA). 3-(2-pyridyldithio) propionyl- dipalmitoyl- phosphatidylethanolamine (DTP DPPE) was prepared by reacting SPDP with DPPE as described by Barbet *et al.* (1981).

*Prepartion of immunoliposomes containing $^{10}B$-compound*

Egg yolk PC (5 μ moles), cholesterol (5 μ moles) and DTP-DPPE (0.25 μ moles) dissolved in chroloform-methanol (2:1) were mixed in a conical flask. The organic solvent was removed by evaporation at 40°C. A half ml of 25, 100, 250 mM $^{10}B$-compound ($Cs_2{}^{10}B_{12}H_{11}SH$) solution and 20 μ l carboxyfluorescein (CF) (2 μ moles) were added to the dried lipid film, and then multilamellar vesicles were prepared by vortex dispersion. Uncapsulated $^{10}B$-compound and CF were removed by washing with centrifugation at 20,000 *g*. After treatment for 30 min at room temperature with 20 mM dithiothreitol to ensure the functional SH-group, the liposome was centrifuged at 20,000 *g*. One ml of monoclonal antibody (0, 0.5, 1.0, 4.0 mg $ml^{-1}$) was incubated with an excess amount of SPDP for 30 min at room temperature. After removal of free SPDP by passage through a Sephadex G 25 column, the liposomes were suspended in the antibody solution. After incubation at 4°C overnight, the boronated immunoliposomes ($^{10}B$-Lip-MoAb) were washed by centrifugation at 20,000 *g* and suspended in 1 ml of 10 mM veronal buffer, pH 7.4, supplemented with 0.4% gelatin. An average diameter of the liposome was estimated to be 4.7 μ in dynamic light scattering analysis.

The liposome prepared was confirmed to be stable in serum as reported (Yeagle, 1985), and boron was confirmed not to leak out of the liposome.

*The determination of $^{10}B$-compound concentration entrapped in liposomes*

The amount of $^{10}B$-compound entrapped in liposomes was determined by a colorimetric method in the presence of curcumine (Ikeuchi & Amano, 1978).

After oxidative degradation with potassium permanganate, boron was extracted with chloroform containing 2-ethyl-1, 3-hexanediol. Boron in the extract was converted into the boron-curcumine complex by adding an acetic acid solution of curcumine. Concentrated sulfuric acid was then added to the solution. After dilution with 95% (v/v) ethanol, the optimal absorbancy of the solution at 554 nm was measured by spectrophotometry.

*Thermal neutron irradiation*

AsPC-1 cells, $5 \times 10^4$ cells/culture, were incubated in a 96 well-microplate at 37°C in 5% $CO_2$ in air for 8 h in the presence of immunoliposomes. After washing, the cells were irradiated with thermal neutron at the TRIGA-II atomic reactor of Rikkyo University (Yokosuka, Japan). After irradiation, 0.25 μ Ci $^3H$ TdR was added to each well and incubated for further 8 h. Then, the cells were harvested and the incorporation of thymidine was estimated in a liquid scintillation spectrometer.

*The determination of gamma-ray dose generated at the thermal neutron irradiation in the thermal column*

The gamma-ray generated during thermal neutron irradiation was measured using the ionisation chamber method (ICRU, 1964) at the irradiation points.

*Gamma-ray irradiation of cells*

AsPC-1 cells were irradiated by gamma-rays from the $^{137}Cs$ source in Gamma Cell 40 (Atomic Energy of Canada, Ottawa, Canada).

### Results

*Reactivity of immunoliposomes to target tumour cells*

In order to examine the reactivity of immunoliposomes to target cells, AsPC-1 cells were incubated with liposomes for 30 min at room temperature, stained with second antibody and examined under a microscope. The reaction was strongly positive, when the cells were incubated with the original or a 1:10 dilution of the liposome preparation. The cells were stained weakly with the 1:100 dilution. Figure 1 shows positive staining of the AsPC-1 cells with 1:10 dilution of the liposome suspension. AsPC-1 cells incubated with liposomes conjugated with anti-DNP were not stained at all. The reactivity of the immunoliposomes to AsPC-1 cells was also confirmed by the use of liposomes with fluorescent dye, carboxyfluorescein (data not shown).

*The concentration of $^{10}B$ entrapped in liposomes*

The immunoliposomes were prepared with 100 mM or 250 mM $^{10}B$-compound and 4 mg $ml^{-1}$ SPDP-anti-CEA. These preparations were assayed for entrapped $^{10}B$. The amounts of $^{10}B$ in immunoliposomes prepared with 100 mM and 250 mM

**Figure 1** The reactivity of immunoliposome to AsPC-1 cells. **a**, anti-CEA MoAb (1:10 dilution); **b**, anti-DNP MoAb (1:10 dilution). The magnification of all photomicrographs is × 800.

$^{10}$B-compound were 178±33 and 623±80 μg ml$^{-1}$ liposome, respectively. Anti-CEA conjugated with liposome was estimated to be 734.0 ± 37 μg ml$^{-1}$ liposome. Thus, the liposomes prepared with 100 mM and 250 mM $^{10}$B compound were calculated to contain $5.0 \times 10^3$ and $1.3 \times 10^4$ $^{10}$B for each antibody, respectively. These results are shown as averages and standard error of the values obtained from five separate assays.

### *Growth inhibition of AsPC-1 cells treated with immunoliposomes*

In order to examine the effect of immunoliposomes on the growth of AsPC-1 cells, the cells were treated with liposomes prepared with 250 mM $^{10}$B-compound and 4 mg ml$^{-1}$ anti-CEA or anti-DNP. After washing to remove free liposomes, the cells were irradiated with various fluences of thermal neutrons and cultured *in vitro*. As shown in Figure 2, AsPC-1 cells treated with the original suspension of immunoliposomes showed a reduction in growth by 50% at $1 \times 10^{12}$ fluence or more of thermal neutrons. When AsPC-1 cells were treated with immunoliposomes prepared with anti-DNP instead of anti-CEA, they grew as well as untreated cells. As shown in Figure 3, the decrement in cell growth was dependent on the dose of the liposomes used, and liposomes without antibody or $^{10}$B-compound exerted little effect on cell growth.

These results indicate that immunoliposomes could carry $^{10}$B-compound to the target cells and exert toxic effects on them.

### *Effect of $^{10}$B-compound concentration in immunoliposomes on cytotoxicity*

In order to confirm the role of $^{10}$B-compound in the immunoliposome, the effect of $^{10}$B-compound concentration on the cytotoxicity of the liposome against AsPC-1 cells was examined. Liposomes were prepared by using 25, 100, 250 mM or without $^{10}$B-compound and conjugated with 4 mg ml$^{-1}$ anti-CEA. These immunoliposomes were examined for their cytotoxic effects on AsPC-1. As shown in Figure 4, the liposomes prepared in 25 mM $^{10}$B-compound showed little effect on AsPC-1 cell growth. However, liposomes prepared with 100 mM or 250 mM $^{10}$B-compound significantly inhibited the growth of the cells and those prepared with $^{10}$B-compound under 100 mM and greater than 25 mM may have recognisable dose-dependent effectiveness.

**Figure 2** Effect of thermal neutrons *in vitro* on AsPC-1 cells treated with $^{10}$B-Immunoliposome (B-Lip-MoAb). The immunoliposomes were prepared by using 250 mM $^{10}$B-compound and 4 mg ml$^{-1}$ anti-CEA or anti-DNP. Cytotoxicity was estimated by $^{3}$H-TdR incorporation. Each point represents the mean±s.e. of triplicate assay. $^{10}$B-Immunoliposome conjugated with anti-CEA (O——O) or anti-DNP (●– – –●). Medium only (X······X) or $^{10}$B solution (500 ppm) (Δ– – –Δ) are also shown.

**Figure 3** Growth inhibiton of AsPC-1 cells treated with $^{10}$B-immunoliposome ($^{10}$B-Lip-MoAb). AsPC-1 cells were treated with various concentrations of liposomes prepared with 250 mM $^{10}$B-compound and conjugated with 4 mg ml$^{-1}$ anti-CEA or treated with plain liposomes, washed and irradiated with thermal neutrons. AsPC-1 cell growths treated with undiluted (O——O), diluted 1:100 (Δ– – –Δ) or 1:1000 diluted (□– – –□) $^{10}$B-Lip-MoAb, and also with undiluted (●——●), 1:1000 diluted (▲– – –▲) or 1:1000 diluted (■– – –■) plain liposomes are shown. The growth of irradiated cells without liposome treatment (X······X) is also shown.

**Figure 4** Effect of $^{10}$B-compound concentration in immunoliposomes on cytotoxicity. The growth of AsPC-1 cells treated with liposomes prepared with 25 (▲——▲), 100 (□– – –□), 250 mM (O——O) or without $^{10}$B-compound (●– – –●) and conjugate with 4 mg ml$^{-1}$ of anti-CEA and irradiated with thermal neutrons is shown. The growth of irradiated cells without liposome treatment (X·····X) is also shown.

These results indicate that the immunoliposomes must be prepared with a concentration of 100 mM or more of $^{10}$B-compound for effective BNCT under our experimental conditions.

### *Effect of antibody density on the cytotoxicity*

Antibody on the liposomes plays a role in their carriage to target cells. In the next experiments, therefore, liposomes prepared with 250 mM $^{10}$B-compound were conjugated with various concentrations of anti-CEA, and the effects of these immunoliposomes on AsPC-1 cell growth were examined after thermal neutron irradiation. As shown in Figure 5, AsPC-1 cells showed reduced growth at $1 \times 10^{12}$ n cm$^{-2}$ and more of thermal neutron fluences as the increment of the antibody concentration used for the preparation of immunoliposomes. These results indicate that the antibody used for

**Figure 5** Effect of antibody density on liposomes on cytotoxicity. Liposomes (Lip) prepared with 250 mM $^{10}$B-compound were conjugated with 0.5 mg ml$^{-1}$ (▲——▲), 1.0 mg ml$^{-1}$ (□— — —□), 4.0 mg ml$^{-1}$ (O——O) or without (●— — —●) anti-CEA. The effects of these immunoliposomes ($^{10}$B-Lip-MoAb) on AsPC-1 cell proliferation were examined after thermal neutron irradiation. The growth of irradiated cells without liposome treatment (X·····X) is also shown.

the preparation of immunoliposomes plays an essential role in targeting the liposomes to tumour cells.

*Effect of gamma-rays generated by thermal neutrons*

There is a possibility that the inhibition of AsPC-1 cells described above was actually due to the gamma-rays generated by thermal neutrons. As shown in Table I, cells irradiated with thermal neutrons were also irradiated with various doses of gamma-rays generated depending on the neutron dose. These doses of gamma-rays did not exert any inhibitory effect on AsPC-1 cell growth. With 3.36 Gy gamma-rays, growth of AsPC-1 cell was suppressed weakly but significantly.

*Failure of inhibition of tumour cell growth with soluble* $Cs_2{}^{10}B_{12}H_{11}SH$

In order to examine the effects of soluble $^{10}$B-compound ($Cs_2$ $^{10}$B H SH) on the proliferation of AsPC-1 cells, the cells were suspended in various concentrations of $^{10}$B-compound solution. After they were irradiated with $1 \times 10^{12}$ to $5 \times 10^{12}$ n cm$^{-2}$ of thermal neutron, their growth was examined. As shown in Table II, the soluble $^{10}$B-compound did not significantly suppress the cell growth even at 2000 ppm (4 mM). If all of the liposomes prepared by using 250 mM of $^{10}$B-compound were lysed to release $^{10}$B-compound into culture medium, it would make 312 ppm $^{10}$B-compound solution.

**Table I** Gamma-ray generated by thermal neutron irradiation and the effect of the irradiation on tumour cell growth

| *Thermal neutron fluences (n cm$^2$)* | *Dose of gamma-ray* (Gy)* | *Cell growth (% uptake of $^3$H-TdR)* | |
|---|---|---|---|
| 0 | 0 | 100 | |
| 5 × $10^{11}$ | 0.258 | 95.3±9.0 | |
| 1 × $10^{12}$ | 0.515 | 93.6±7.0 | |
| 2 × $10^{12}$ | 1.030 | 96.7±18.0 | ($P<0.05$) |
| 5 × $10^{12}$ | 3.360 | 70.6±2.0 | |

*Dose of gamma-ray at neutron irradiation site was measured by the ionisation chamber method. 5 × $10^4$ AsPC-1 cells/200 µl/culture were irradiated by these doses of gamma-ray from $^{137}$Cs source in a separate experiment. After the irradiation, cell proliferation in 8 h was measured by the incorporation of $^3$H-TdR. Results on cell growth are presented as the mean±s.e. in triplicate assays in terms of percentage of $^3$H-TdR uptake of unirradiated AsPC-1 cells.

**Table II** Failure of tumour cell growth inhibiton with soluble $^{10}$B-compound

| *Concentration of $^{10}$B solution (ppm)* | *Thermal neutron fluence (n cm$^2$)* 0 | 1 × $10^{12}$ | 2 × $10^{12}$ | 5 × $10^{12}$ |
|---|---|---|---|---|
| 0 | 57264 ±7595 | 53384 ±1645 | 50044 ±450 | 36895 ±2852 |
| 500 | 58843 ±1922 | 48353 ±1740 | 49977 ±2095 | 36717 ±3646 |
| 1000 | 51707 ±2274 | 49702 ±2624 | 47559 ±5690 | 35661 ±1375 |
| 2000 | 52832 ±3440 | 50528 ±3704 | 48184 ±1694 | 36501 ±3115 |

AsPC-1 cells were suspended in various concentrations of soluble $^{10}$B-compound and irradiated with 1 × $10^{12}$ to 5 × $10^{12}$ n cm$^2$ of thermal neutrons and incubated for 8 h. Cell growth was assayed by the incorporation of $^3$H-TdR in the incubation. The results are shown in the mean±s.e. of triplicate assays.

Because the maximum $^{10}$B concentration entrapped in immunoliposomes was 623 ppm, and an equal volume of medium suspending target cells was added, the $^{10}$B-concentration in the medium must be reduced to half. These results may rule out the possibility that soluble $^{10}$B-compound in the medium emits alpha-particles by thermal neutron irradiation thereby injuring the cells.

### Discussion

Antibody reactive to tumour cells is one of the most useful vehicles for ensuring selective accumulation of boron in tumours.

The effect of the $^{10}$B-conjugated antibody on tumour cell growth in BNCT was reported first by Mizusawa *et al.* (1982), Goldenberg *et al.* (1984). They conjugated 50 boron atoms directly to an antibody molecule, but the antibody did not work efficiently in BNCT.

It was estimated that $10^9$ $^{10}$B atoms are required to destroy one tumour cell in BNCT (Alam *et al.*, 1984). When antibody directly conjugated with $^{10}$B-compound was used in BNCT, the quantity of $^{10}$B atom delivered to a cell was proportional to the density of cell surface antigen molecules. According to Alam *et al.*, an antibody has to be conjugated with $10^3$ $^{10}$B atoms to destroy a tumour cell with $10^6$ epitopes on its surface. One thousand, three hundred boron atoms were reported to be conjugated to a molecule of monoclonal antibody by using SPDP (Alam *et al.*, 1985).

A monoclonal antibody against alpha-foetoprotein was found to exert some cytotoxic effect on AH66 tumour cells in BNCT *in vitro* (Takahashi *et al.*, 1987). However, a heavy boronation of antibody has been shown to markedly reduce the antibody reactivity (Alam *et al.*, 1985; Takahashi *et al.*, 1987) and the numbers of epitope on the tumour cell surface have been estimated to be at most $10^6$/cell (Tsukada *et al.*, 1982; Barth *et al.*, 1990). These results indicate that monoclonal antibody directly conjugated with $^{10}$B-compound has limited application in BNCT.

A liposome is a vesicle which could entrap various materials, and a method of conjugating protein molecules on the surface by SPDP was developed (Barbet *et al.*, 1981). Therefore, it is possible for liposomes to carry a large amount of substance to the target cell surface, if the substance is entrapped in the liposome conjugated with monoclonal antibody specific against the cells. In fact, liposome-bearing antibody against human $\beta_2$-microglobulin was found to bind specifically to human cells but not to murine cells (Leserman *et al.*, 1981). Immunoliposomes containing actinomycin D were reported to exert cytotoxic effect in mammary carcinoma cells in an experimental model (Hashimoto *et al.*, 1983).

In experiments reported here, immunoliposomes suppressed tumour cell growth *in vitro* after thermal neutron irradiation.

Suppression was dependent upon the concentration of entrapped $^{10}$B-compound and also upon the density of anti-CEA conjugated with liposome. These results in *in vitro* experiments suggest that an immunoliposome containing $^{10}$B-compound could be applied in BNCT as an effective carrier of $^{10}$B-compound to target cells, and the evaluation of the system in *in vivo* experiments remains to be carried out. In the present experiment, gamma-rays generated by thermal neutron irradiation did not exert toxic effects on cell growth at less than 3.36 Gy. Therefore, $1 \times 10^{12}$ or $2 \times 10^{12}$ n cm$^{-2}$ flux of thermal neutron may be recommended for BNCT, because inhibition of cell growth by concomitant gamma-rays was found to be negligible at these doses. The use of a more powerful atomic reactor may permit generation of more fluxes of thermal neutrons with less gamma-rays.

In the present experiments, immunoliposomes were found to be potential tools for BNCT, but problems remain to be solved before application to *in vivo* BNCT. First, lipid components of liposomes should be studied more extensively. In the present experiment, multilamellar liposomes were used, since small unilamellar ones composed of our lipid component were shown to be unstable in culture, although small unilamellar liposomes could be suitable for BNCT. If heat-sensitive liposomes (Sullivan & Huang, 1985) were used for BNCT, treatment with hyperthermia could possibly be combined with BNCT to improve the efficiency of targeting of $^{10}$B to tumour cells. Secondly, the technique of conjugating antibody to liposome has to be improved, since F(ab')$^2$ fragments seem to be better for targeting (Martin *et al.*, 1981), because depletion of the Fc fragment of the antibody molecule can obviate trapping of the molecule by Fc receptors on phagocytes or other cells.

However, the most important problem to resolve before clinical application of BNCT with immunoliposomes is how to carry the liposome specifically to the target tumour, or to promote penetration of blood vessels to reach tumour cells. It is conceivable that a complement component or some other substance could be conjugated to promote permeability of small blood vessels.

The authors are grateful to Dr Alistair Renwick, Department of Biochemistry, University of Auckland, Auckland, New Zealand, for criticism of the manuscript.

The work was supported in part by a Grant-in-Aid from the Ministry of Education, Science and Culture (No. 01570741).

### References

ALAM, F., SOLOWAY, A.H., BARTH, R.F., JOHNSON, C.W. & CAREY, W.E. (1984). Boronation of polyclonal and monoclonal antibodies for neutron capture. In: *First International Symposium on Neutron Capture Therapy*, Cambridge, MA, October 12–14, 1983. *Brookhaven Natl. Lab. Report*, 51730, 229.

ALAM, F., SOLOWAY, A.J., MCGUIRE, J.E., BART, R.F., CAREY, W.E. & ADAMS, D. (1985). Dicesium N-succinimidyl 3-(undecahydro-closo-dodecaboranyldithio) propionate, a novel heterobifunctional boronating agent. *J. Med. Chem.*, **28**, 522.

BANGHAM, A.D., STANDISH, M.M. & WATKINS, J.C. (1965). Diffusion of univalent ions across the lamellae of swollen phospholipids. *J. Mol. Biol.*, **13**, 238.

BARBET, J., MACHY, P. & LESERMAN, L.D. (1981). Monoclonal antibody covalently coupled to liposomes specific targeting to cells. *J. Supramolecular Structure and Cellular Biochemistry*, **16**, 243.

BARTH, R.F., SOLOWAY, A.H. & FAIRCHILD, R.G. (1990). Boron neutron capture therapy of cancer. *Cancer Res.*, **50**, 1061.

CHEN, W.H., HOROSZEWICZ, J.S., LEONG, S.S. & 7 others (1982). Human pancreatic adenocarcinoma: *in vitro* and *in vivo* morphology of a new tumor line established from ascites. *In vitro*, **18**, 24.

GOLD, P. & FREEDMAN, O.S. (1965). Demonstration of tumor-specific antigens in human colonic carcinomata by immunological tolerance and absorption technique. *J. Exp. Med.*, **121**, 439.

GOLDENBERG, D.M., SHARKEY, R.M., PRIMUS, F.J., MIZUSAWA, E. & HAWTHORNE, M.F. (1984). Neutron-capture therapy of human cancer: *in vivo* results on tumor localization of boron-10 labeled antibodies to carcinoembryonic antigen in the GW-39 tumor model system. *Proc. Natl Acad. Sci. USA*, **81**, 560.

HASHIMOTO, Y., SUGAWARA, M., MASUKO, T. & HOJO, H. (1983). Antitumor effect of actinomycin D entrapped in liposomes bearing subunits of tumor-specific monoclonal immunoglobulin M antibody. *Cancer Res.*, **43**, 5328.

HSU, S.M., RAINE, L. & FANGER, H. (1981). Use of avidin-biotin-peroxidase complex (ABC) in immunoperoxidase techniques: a comparison between ABC and unlabeled antibody (PAP) procedures. *J. Histochem. Cytochem.*, **29**, 577.

HATANAKA, H. (1986). *Boron-Neutron Capture Therapy for Tumors*. Nishimura Co. Ltd: Niigata.

ICRU REPORT No.10.b (1964). *Physical Aspects of Irradiation*, NBS Handbook, 85, 1.

IKEUCHI, I. & AMANO, T. (1978). A colorimetric determination of boron in biological materials. *Chem. Pharm. Bull.*, **26**, 2619.

KLEIST, S., CHAVANEL, G. & BURTIN, P. (1972). Identification of an antigen from normal human tissue that crossreacts with the carcinoembryonic antigen. *Proc. Natl Acad. Sci. USA*, **69**, 2492.

KONNO, H., SUZUKI, H., TADAKUMA, T. & 9 others (1987). Antitumor effect of adriamycin entrapped in liposomes conjugated with anti-human fetoprotein monoclonal antibody. *Cancer Res.*, **47**, 4471.

KRUGER, P.G. (1940). Some biological effects of nuclear disintegration products on neoplastic tissue. *Proc. Natl Acad. Sci. USA*, **26**, 181.

LAEMMLI, U.K. (1970). Cleavage of structural proteins during the assembly of the head of bacteriophage T4. *Nature*, **277**, 680.

LESERMAN, L.D., MACHY, P. & BARBET, J. (1981). Cell-specific drug transfer from liposomes bearing monoclonal antibodies. *Nature*, **293**, 226.

LOCHER, G.L. (1936). Biological effects and therapeutic possibilities of neutrons. *Amer. J. Roentgenol. Radium Ther.*, **36**, 1.

MARTIN, J.F., HUBBEL, W.L. & PAPAHADJOPOULOS, D. (1981). Immunospecific targeting of liposomes to cells: a novel and efficient method for covalent attachment of Fab fragments via disulfide bonds. *Biochemistry*, **20**, 4229.

MISHIMA, Y., ICHIHASHI, M., NAKANISHI, T. & 4 others (1983). Cure of malignant melanoma by single thermal neutron capture treatment using melanoma seeking compounds: B/melanogenesis interaction to *in vitro/in vivo* radiobiological analysis to preclinical studies. *Proceedings of First International Symposium on Neutron Capture Therapy*. Brookhaven National Laboratory Report No. 51730, 355.

MISHIMA, Y., HONDA, C., ICHIHASHI, M. & 7 others (1989). Treatment of maligant melanoma by single thermal neutron capture therapy with melanoma-seeking $^{10}$B-compound. *Lancet*, **ii**, 388.

MIZUSAWA, E., DAHLMAN, H.L., BENNETT, S.J., GOLDENBERG, D.M. & HAWTHORNE, M.F. (1982). Neutron-capture therapy of human cancer: *in vitro* results on the preparation of boron-labeled antibodies to carcinoembryonic antigen. *Proc. Natl Acad. Sci. USA*, **79**, 3011.

SULLIVAN, S.M. & HUANG, L. (1985). Preparation and characterization of heat-sensitive immunoliposomes. *Biochim, Biophys. Acta*, **812**, 116.

SWEET, W.H. (1951). The uses of nuclear disintegration in the diagnosis and treatment of brain tumor. *New England J. Med.*, **245**, 875.

TAKAHASHI, T., FUJII, Y., FUJII, G. & NARIUCHI, H. (1987). Preliminary study for application of anti-alpha-feto-protein monoclonal antibody to boron-neutron capture therapy. *Jpn. J. Exp. Med.*, **57**, 83.

TANAKA, T., SUZUKI, S., MASUKO, T. & HASHIMOTO, Y. (1989). In vitro targeting and cytotoxicity of adriamycin in liposomes bearing monoclonal antibody against rat or human gp125 cell proliferation-associated antigen. *Jpn. J. Cancer Res.*, **80**, 380.

TSUKADA, Y., BISCHOF, W.K-D., HIBI, N. & 3 others (1982). Effect of a conjugate of daunomycin and antibody to rat α-fetoprotein-producing tumor cells. *Proc. Natl Acad. Sci. USA*, **79**, 621.

YEAGLE, P.L. (1985). Cholesterol and the cell membrane. *Biochim. Biophys. Acta.*, **822**, 267.